# Clinicopathological parameters associated with cervical lymph node metastases in differentiated thyroid cancer

**DOI:** 10.5152/eurasianjmed.2024.23182

**Published:** 2024-06-01

**Authors:** Hayriye Tatlı Doğan, Aydan Kılıçarslan, Ayça Dilşad Çağlayan, Nuran Sungu

**Affiliations:** 1 Department of Pathology, Ankara Yıldırım Beyazıt University Faculty of Medicine, Ankara City Hospital, Ankara, Türkiye; 2 Department of Pathology, Ankara Etlik City Hospital, Ankara, Türkiye

**Keywords:** Cervical lymph node, differentiated thyroid cancer, metastasis

## Abstract

**Background::**

Lymph node metastasis (LNM) has an important role for the prognosis of differentiated thyroid cancer (DTC). The aim of the study was to investigate the effect of clinicopathologic parameters on cervical LNM in DTC.

**Methods:**

The patients who underwent thyroidectomy along with neck dissection were analyzed retrospectively.

**Results:**

Of the 150 patients diagnosed with DTC who underwent neck dissection, 1 had follicular thyroid carcinoma and 149 had papillary thyroid carcinoma (PTC). The median tumor size was 14.0 mm. The tumor diameter with the highest specificity and sensitivity for the detection of LNM was ≥11.5 mm**. **Extrathyroidal extension (ETE) was observed in 35.3% of the patients. The rate of multifocality in tumors with extrathyroidal spread was significantly higher than in tumors without ETE. LNM was observed in 60.0% of the patients. ETE was present in 28.9% of the tumors that had LNM. Lymphovascular invasion (LVI), perineural invasion (PNI), and positive surgical margin were observed in 13.3%, 2.7%, and 14% of the patients respectively. A significant positive correlation was found between LNM and tumor diameter, ETE, positive tumor margin and LVI (*Pp* = .006, *P* = .031, *P *= .002, and *P* = .014, respectively).

**Conclusion:**

In this study, ETE, LVI, positive tumor margin, and tumor diameter greater than 11.5 mm were significantly correlated with the presence of LNM. These findings may be useful in bringing to mind the possibility of lymph node metastases that have not been able to be detected before surgery and in monitoring these patients more closely.

**Main Points**

- Thyroid cancer is the most common endocrine malignancy.
- Differentiated thyroid cancers constitute ninety percent of thyroid cancers.
- Lymph node metastasis is a well-known prognostic factor.
- Tumor diameter ≥11.5 mm is found with the highest specificity and sensitivity for the detection of LNM.

## Introduction

Thyroid cancer is the most common endocrine malignancy.^1^ Ninety percent of thyroid cancers are differentiated thyroid cancer(DTC). Cervical lymph node metastases (LNM) are observed in 20-50% of patients with DTC. LNM is an especially important prognostic parameter in papillary thyroid cancer (PTC) that is associated with an increased recurrence rate and requires additional radioactive iodine treatment or surgery. It also leads to a relative increase in the risk of death^2^ related to PTC. It is important to determine whether clinicopathological parameters such as age, sex, tumor diameter, tumor location, bilaterality, and multifocality contribute to predicting LNM in DTC. Performing neck and thyroid examinations alongside anamnesis in patients who present to family medicine outpatient clinics, which are primary health care centers, for any reason whatsoever is of great importance for early detection of the suspicious nodules that are frequently observed in the population. However, patients should be directed to more comprehensive centers with endocrinologists, radiologists, pathologists, and surgeons in terms of follow-up, treatment, and surgical decision for these nodules.

Although detailed radiological and physical examination of cervical lymph nodes is performed before surgery in patients diagnosed with PTC, occult metastases may not be detected radiologically or clinically. In addition, fine needle aspirations performed before surgery to determine the presence of metastasis in enlarged and suspicious lymph nodes may not be diagnostic. Since occult metastases can be overlooked, patients may present with lymph node or distant organ metastases a few years after thyroidectomy. Predictive clinical and histopathological parameters are needed for additional treatment choices for the patient. Thus, in this study we aimed to investigate the predictive role of the clinicopathological findings for the detection of LNM. in DTC patients.

## Material and Methods

All patients who underwent neck dissection with thyroidectomy between 2009 and 2017 were analyzed retrospectively. Detailed information about tumors and patients was obtained from pathology reports and the hospital database. The patients were evaluated for the weight of the thyroid, the localization, type, size, bilaterality/multifocality of the tumor, the presence of ETE, LVI, PNI, positive surgical margin, LNM, and extracapsular extension in the metastatic lymph node. The study protocol conforms to the Declaration of Helsinki and was approved by the Ankara Yildirim Beyazit University Faculty of Medicine (decision number: 26379996/132; date: May 7, 2018). Written informed consent was obtained from the patients who agreed to take part in the study.

### Statistical Analysis

Descriptive statistics, such as age, thyroid weight, tumor diameter, and the number of LNMs, were expressed as median IQR (interquartile range), and categorical variables were expressed as n (%). The Mann–Whitney *U* test was used to compare age, thyroid weight, and tumor diameter according to sex and the presence of LNM. Cross tabulations were created to examine the comparative distribution of categorical variables. The chi-squared (likelihood ratio) test was used to compare the rates of tumor focus distribution according to sex and ETE.

ROC analysis was performed to predict the minimum tumor diameter value associated with the presence of LNM.

Sensitivity, selectivity, positive predictive value (PPV), negative predictive value (NPV), overall accuracy ratio, likelihood ratio+ (LR+), and likelihood ratio− (LR−) values was calculated for different cut-off points obtained from the ROC analysis.

For statistical analysis and calculations, MS Excel 2010 and IBM SPSS Statistics 22.0 (IBM Corp. Released 2013. IBM SPSS Statistics for Windows, Version 22.0. (IBM SPSS Corp.; Armonk, NY, USA) were used. A *P* value of < .05 was considered statistically significant.

## Results

Of the 150 patients diagnosed with DTC, 119 (79.0%) were female and 31 (21%) were male. The median age of the females was 44 years which was significantly lower than the males (*Pp* = .040). The median of the thyroid gland weight in the female patients was significantly less than that in the male patients (*P *= .001). Tumor sizes ranged between 1 and 55 mm with a median of 14.0 mm. The tumor diameter of patients with LNM was significantly larger compared to those of patients without LNM (*P *= .006).

Of the tumors examined, 78 (52.0%) were unifocal and 72 (48%) were multifocal. Unifocal tumors were seen in 52.1% (n = 62) of the female patients and in 51.6% (n = 16) of the male patients.

A total of 239 tumor foci were found in 150 patients. Of these foci, 114 (47.7%) were located in the right lobe, 92 (38.5%) were in the left lobe, and 33 (13.8%) were in the isthmus. Further, 62.8% (n = 49) of the unifocal tumors were in the right lobe, 34.6% (n = 27) in the left lobe, and 2.6% (n = 2) in the isthmus.

Among the tumor cases, 1 case was follicular carcinoma and 149 were PTC. Of the PTC cases, 54 (36.2%) were classical variant, 38 (25.5%) were papillary microcarcinoma (PMC), 35 (23.5%) were PTC accompanied by PMC, and 22 (14.8%) were in other groups (other variants of PTC).

Sixty-nine of 150 cases (46.0%) were unilateral, 43 (28.7%) were bilateral, 10 (6.7%) were multifocal, and 28 (18.7%) were bilateral and multifocal. Commonly, classical PTC, PMC, and other types were unilateral (63.0%, n = 34; 57.9%, n = 22; and 50.0%, n = 11, respectively). Nineteen (54.3%) of 35 PTCs accompanied by PMC were bilateral, while 34.3% (n = 12) of these cases were bilateral and multifocal.

35.3% (n = 53n = 53) of the tumors had ETE. Moreover, 62.3% (n = 33) of the tumors showing ETE were multifocal, but this rate was lower in the tumors without ETE (40.2%; n = 39) (*p* = .01).

Twenty (13.3%) patients had LVI and 4 (2.7%) had PNI. Positive surgical margin was found in 21 of 150 cases (14.0%).

While LNM was observed in 60.0% (n = 90n = 90) of the tumors examined, at least 1 and up to 46 metastatic lymph nodes were observed in the tumors with LNM, and the median LNM was 3.0 (IQR = 6.0). Extracapsular extension was present in 28.9% (n = 26) of the tumors with LNM. The association between the presence of LNM and the clinicopathologic data is shown in Table 1.

**Figure 1.**
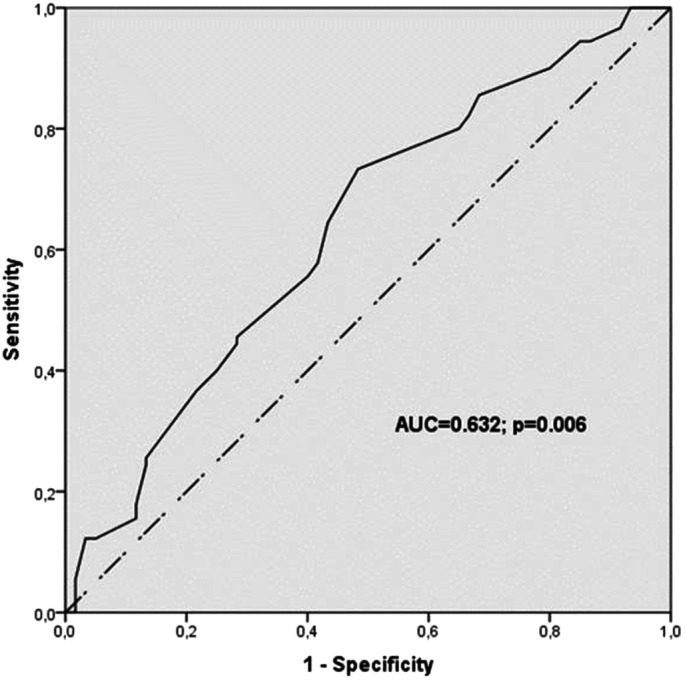
Tumor diameter ROC curve to determine lymph node metastasis.

According to ROC analysis, tumor diameter can be used to detect LNM (AUC = 0.632; *P* = .006) (Figure 1). Sensitivity, selectivity, PPV, NPV, overall accuracy, LR+, and LR– values for different cut-off points were calculated and are presented in Table 2.

The values in Table 2 indicate that the sensitivity (rate of distinguishing true cases) of a tumor diameter of ≥7.5 mm was 90.0% and the selectivity (rate of distinguishing true healthy patients) of this parameter was 20.0%. The acceptable cut-off point with the highest sensitivity was a tumor diameter of ≥7.5 mm, while the acceptable cut-off point with the highest selectivity was a tumor diameter of ≥25.0 mm. The cut-off point with the highest overall accuracy of 64.7% in terms of sensitivity and selectivity was a tumor diameter of ≥11.5 mm. Moreover, the cut-off point of a tumor diameter of ≥11.5 mm had a sensitivity of 73.3% and selectivity of 51.7% for detecting LNM (Figure 1).

## Discussion

Various clinicopathological parameters such as tumor diameter, ETE, LNM, and distant metastasis are well-known prognostic factors in patients with PTC.^1^ Age and sex are also known prognostic parameters. Shi et al^3^ subdivided DTC patients into subgroups of 10-year age ranges and found that DTC in elderly patients generally showed more aggressiveness and that cancer-specific survival was significantly lower in patients older than 60 years of age. They highlighted that male sex was also an important indicator of poor prognosis. In the current study, no relationship between age and LNM or sex and LNM was found since the study did not group patients according to age, which can be considered a limitation. However, the age of male DTC patients with LNM was higher than that of female DTC patients with LNM. Due to the effect of age and sex on prognosis, it would be appropriate for clinicians to be aware of the poor prognosis of these patients and to follow them up more frequently.

The universally used TNM staging system proposes patients be divided into risk groups of tumor sizes of <1 cm, 1-4 cm, and >4 cm.^4^ In prior studies, it has been reported that the disease-specific mortality rate is significantly higher in tumors larger than 40 mm.^5^ In the current study, a positive correlation between tumor diameter and LNM was also found.

In addition, in some studies patients with DTCs larger than 1 cm had more frequent ETE, LVI, and LNM than those with DTCs smaller than 1 cm.^6^ Wang et al^7^ also found a significant relationship between tumor diameters larger than 1 cm and LNM. In the current study, a tumor diameter of ≥11.5 mm had the highest specificity and sensitivity for determining LNM.

The rate of LNM in PTC varies between 20% and 90%.^8^ Sun et al^9^ found a lymph node metastasis rate of 65.71% in patients with DTC who underwent cervical lymph node dissection. In the present study, 60% of the examined patients had LNM.

Recent evidence suggests that microscopic ETE is associated with reduced disease-free survival, independent of tumor size.^10^ In their study that included 1092 patients with solitary papillary TMC, Yin et al^11^ found a significant relationship between central LNM and ETE. In the present study, a positive significant relationship was found between ETE and cervical LNM (*P* < .05).

PTC tends to spread lymphatically. Thus, lymphatic invasion should be noted in pathology reports since it is an indicator of an aggressive invasion pattern. In their meta-analysis, Hui Qu et al^12^ found a significant relationship between LVI and central LNM. In the current study, a significant relationship was found between LVI and LNM.

The “margin” is defined as the outer surface of the thyroid sample and/or the inked tissue edge of the sample. Margin status is determined by assessment of the relationship between the inked edge of the tissue and the tumor.^13^ It should be noted that the thyroid capsule is not an anatomically defined structure. Prior studies have shown microscopically that the thyroid capsule is focally deficient or absent in the majority of thyroid glands evaluated at autopsy.^14^ Studies on the prognostic value of close margins are limited in number.^13^ In the current study, a significant correlation between margin positivity and LNM was found.

Clinically, the presence of two or more anatomically separated foci in the thyroid gland is considered as multifocal PTC. Studies have reported multifocality rates varying between 18% and 87%.^15^ In our study, the multifocality rate of the patients evaluated was 48%. Multifocal PTCs are common and have more aggressive behavior and a poorer prognosis. Wang et al^7^ found a significant positive correlation between LNM and multifocal tumors.

In the current study, no correlation was found between multifocality and LNM. However, most of the tumors with ETE (62.3%) were multifocal, which was a statistically significant result.

Our study has some limitations. The first is the retrospective and single-centered nature of the study. Second is the limited number of cases examined. This can be explained by the fact that although prophylactic central lymph node dissection is still controversial, central lymph node dissection is not routinely performed in every patient undergoing thyroidectomy and therefore the number of cases of thyroidectomy with lymph node dissection is limited.^12^ The third limitation of the study is the lack of genetic mutation information of the patients. Adding genetic alterations related with prognosis and recurrence such as RET, RAS, and BRAF mutations^16^ to histological parameters will be more effective in the management of these patients.

In the present study, a significant correlation was found between LNM and clinicopathological parameters such as tumor diameter, ETE, LVI, and positive tumor margin. Moreover, the tumor diameter with the highest specificity and sensitivity for LNM detection was ≥11.5 mm. Lesions larger than 11.5 cm radiologically or with intraoperative suspicion of extrathyroidal extension may guide surgeons to perform prophylactic central lymph node dissection besides thyroidectomy in terms of the possibility of lymph node metastasis.

In addition, postoperative findings in thyroidectomy specimens including LVI, microscopic ETE, positive tumor margin may be predictive for LNM. These patients should be followed more closely for lymph node or distant metastases.

## Figures and Tables

**Table 1.** Relationship Between the Status of Lymph Node Metastasis and Clinicopathological Data

Variables	LNM		Test Statistics
	Absent	Present	
	Median (IQR)	Median (IQR)	*Z*; *P*
Age (year)	47.0 (23.0)	44.0 (17.0)	*Z* = 0.424; *P *= .672
Thyroid weight (g)	22.0 (18.0)	22.0 (14.0)	*Z* = 0.622; *P* = .534
Tumor diameter (mm)	11.0 (9.0)	15.0 (13.0)	*Z* = 2.752; ***P *= .006**
	n (%)	n (%)	*χ* ^2^; *P*
Gender			
Female	52 (43.7%)	67 (56.3%)	*χ* ^2^ = 3.280; *P* = .070
Male	8 (25.8%)	23 (74.2%)	
Multifocality			
Absent	36 (46.2%)	42 (53.8%)	*χ* ^2^ = 2.564; *P* = .109
Present	24 (33.3%)	48 (66.7%)	
Tumor type			
Papillary	59 (39.6%)	90 (60.4%)	*χ* ^2^ = 1.843; *P* = .175
Follicular	1 (100.0%)	0 (0.0%)	
PTC type			
Classical	19 (35.2%)	35 (64.8%)	*χ* ^2^ = 4.131; *P* = .248
Microcarcinoma	20 (52.6%)	18 (47.4%)	
Classical with microcarcinoma	11 (31.4%)	24 (68.6%)	
Other	9 (40.9%)	13 (59.1%)	
Tumor location			
Unilaterality	30 (43.5%)	39 (56.6%)	*χ* ^2^ = 0.644; *P* = .422
Bilaterality/multifocality	30 (37.0%)	51 (63.0%)	
ETE			
Absent	45 (46.4%)	52 (53.6%)	*χ* ^2^ = 4.673; *P* = .031
Present	15 (28.3%)	38 (71.7%)	
LVI			
Absent	57 (43.8%)	73 (56.2%)	*χ* ^2^ = 6.010; *P* = .014
Present	3 (15.0%)	17 (85.0%)	
PNI			
Absent	59 (40.4%)	87 (59.6%)	*χ* ^2^ = 0.409; *P* = .522
Present	1 (25.0%)	3 (75.0%)	
Surgical margin			
Negative	58 (45.0%)	71 (55.0%)	*χ* ^2^ = 9.450; *P* = .002
Positive	2 (9.5%)	19 (90.5%)	

*P* < .05 was accepted as statistically significant.

ETE, extrathyroidal extension; LNM, lymph node metastasis; LVI, lymphovascular invasion; PNI, perineural invasion; PTC, papillary thyroid carcinoma.

**Table 2.** Sensitivity and Selectivity Values for Different Cut-off Points of Tumor Diameter

Test Criteria	Tumor Diameter			
	7.5 mm	11.5 mm	20.0 mm	25.0 mm
Sensitivity	90.0	73.3	36.7	24.4
Selectivity	20.0	51.7	78.3	86.7
PPV	62.8	69.5	71.7	73.3
NPV	57.1	56.4	45.2	43.3
General accuracy %	62.0	64.7	53.3	49.3
LR+	1.1	1.5	1.7	1.8
LR−	0.5	0.5	0.8	0.9

LR, likelihood ratio; NPV, negative predictive value; PPV, positive predictive value.
